# Arthur Bernard Cridland

**Published:** 1934

**Authors:** 


### Obituary

---

### ARTHUR BERNARD CRIDLAND, F.R.C.S.

Arthur Bernard Cridland was educated at Clifton College, whence he came to the Bristol Medical School in October, 1891. An extremely attractive personality, he soon became one of the most popular men of his time : a steady worker and a hard player. For some years he played football for the Clifton Football Club, and later hockey for the Royal Infirmary. When, in 1891, the late W. C. Swayne made his record recruiting campaign Cridland was one of the first to join, and went into No. 6 Company of the old Gloucester Volunteer Artillery. Here his keenness and efficiency soon distinguished him, his shooting and drill prizes were numerous, and for some years he always acted as layer at gun practice. He was one of the detachment from the Regiment which lined London Bridge during the Diamond Jubilee procession in 1897.

But outside the Medical School he was best known as an amateur actor and a staunch supporter of the Bristol Medical Dramatic Club. He made his first appearance under the management of James Swain, and in the following year played the lead for D. S. Davies's first essay in production—the best juvenile lead the club ever had. He soon attracted the notice of Frank Morris, and was enrolled in his famous band of amateurs.

During his time at the Royal Infirmary Cridland came under the influence of two men who had a far-reaching effect on his future, Greig Smith and Richardson Cross, and determined to make eyes his life's work. After completing his medical education at St. Mary's Hospital, he returned to the Bristol Eye Hospital as house-surgeon, and while there was asked by Paul Bush to join the staff of Princess Christian's Hospital. Soon after his return from South Africa Cridland left Bristol for Wolverhampton, where his life's work was done : he was appointed Ophthalmic Surgeon to the Wolverhampton Royal Hospital and the Staffordshire General Infirmary, and

Surgeon to the Wolverhampton and Midland Counties Eye Infirmary. He became a recognized authority on injuries to the eye, and was elected in 1914 Secretary to the Ophthalmological Congress, a post which he filled until 1929, when he was elected Master of the Congress.

He was Vice-President of the Council of British Ophthalmologists and of the Ophthalmological Society of the United Kingdom, President of the Midlands Ophthalmological Society, and a member of the French Ophthalmological Society. He died on 29th June, 1934, at Wolverhampton, at the age of sixty, leaving a widow and two sons, to whom we offer our deepest sympathy.

